# Lymphoma InterVEntion (LIVE) – patient-reported outcome feedback and a web-based self-management intervention for patients with lymphoma: study protocol for a randomised controlled trial

**DOI:** 10.1186/s13063-017-1943-2

**Published:** 2017-04-28

**Authors:** Lindy P. J. Arts, Lonneke V. van de Poll-Franse, Sanne W. van den Berg, Judith B. Prins, Olga Husson, Floortje Mols, Angelique V. M. Brands-Nijenhuis, Lidwine Tick, Simone Oerlemans

**Affiliations:** 1 Department of Research, Netherlands Comprehensive Cancer Organisation, PO Box 19079, 3501 DB Utrecht, the Netherlands; 2 Division of Psychosocial Research and Epidemiology, Netherlands Cancer Institute, Amsterdam, the Netherlands; 3 CoRPS – Center of Research on Psychology in Somatic Diseases, Department of Medical and Clinical Psychology, Tilburg University, Tilburg, the Netherlands; 4 Department of Medical Psychology, Radboud University Medical Center, Nijmegen, the Netherlands; 5 Department of Internal Medicine, Catharina Hospital Eindhoven, Eindhoven, the Netherlands; 6 Department of Internal Medicine, Máxima Medical Centre, Veldhoven, the Netherlands

**Keywords:** Lymphoma, Intervention, Self-management, Psychological distress, Information provision, Patient-reported outcomes, PRO feedback, Population-based, Randomised controlled trial

## Abstract

**Background:**

Patients with lymphoma are at risk of experiencing adverse physical and psychosocial problems from their cancer and its treatment. Regular screening of these symptoms by the use of patient-reported outcomes (PROs) could increase timely recognition and adequate symptom management. Moreover, self-management interventions intend to enhance knowledge and skills and empower patients to better manage their disease and related problems. The objective of the Lymphoma InterVEntion (LIVE) trial is to examine whether feedback to patients on their PROs and access to a web-based, self-management intervention named *Living with lymphoma* will increase self-management skills and satisfaction with information, and reduce psychological distress.

**Methods/design:**

The LIVE randomised controlled trial consists of three arms: (1) standard care, (2) PRO feedback, and (3) PRO feedback and the *Living with lymphoma *intervention. Patients who have been diagnosed with Hodgkin lymphoma, non-Hodgkin lymphoma, including chronic lymphocytic leukaemia, as registered in the Netherlands Cancer Registry in various hospitals will be selected for participation. Patients are invited via their haemato-oncologist 6 to 15 months after diagnosis. The PRO feedback includes a graphical overview of patients’ own symptom and functioning scores and an option to compare their scores with those of other patients with lymphoma and a normative population of the same age and sex. The *Living with lymphoma *intervention is based on cognitive behavioural therapy components and includes information, assignments, assessments, and videos. Changes in outcomes from baseline to 16 weeks, 12, and 24 months post intervention will be measured. Primary outcomes are self-management skills, satisfaction with information, and psychological distress. Secondary outcomes are health-related quality of life, illness perceptions, fatigue, and health care use.

**Discussion/design:**

The results of the LIVE trial will provide novel insights into whether access to PRO feedback and the *Living with lymphoma *intervention will be effective in increasing self-management skills and satisfaction with information, and reducing distress. The LIVE trial is embedded in a population-based registry, which provides a unique setting to ascertain information on response, uptake, and characteristics of patients with lymphoma in web-based intervention(s). When effective, PRO feedback and *Living with lymphoma *could serve as easily and widely accessible interventions for coping with lymphoma.

**Trial registration:**

Netherlands Trial Register, identifier NTR5953. Registered on 14 July 2016.

**Electronic supplementary material:**

The online version of this article (doi:10.1186/s13063-017-1943-2) contains supplementary material, which is available to authorized users.

## Background

Due to advances in treatment, the 20-year prevalence of Hodgkin lymphoma (HL) and non-Hodgkin lymphoma (NHL) in the Netherlands is expected to increase by 5% to 6300 and 32,000 patients in 2020, respectively [1, 2]. As a result of their cancer and its treatment, patients with lymphoma are at risk of experiencing adverse physical and psychosocial problems, such as fatigue, neuropathy, and cognitive and emotional problems [3–6]. Patients who report adverse problems have a lower health-related quality of life (HRQoL) and visit their physician more often [7–9]. In addition, up to a quarter of patients with lymphoma experience persistent levels of anxiety, depressive feelings, and fears, also called psychological distress [7, 10].

Patient-reported outcomes (PROs) intend to evaluate the impact of a disease and its treatment from the perspective of the patient [11]. PROs are being increasingly recognised to be important in daily practice [12, 13]. Regular screening of physical and psychosocial symptoms by the use of PROs could increase awareness and recognition of symptoms and can contribute to adequate symptom management [11, 14–17]. Moreover, the greater the resources available for coping with symptoms and stress, the lower the risk for psychological distress [18]. Interventions using cognitive behavioural therapy (CBT) components, such as psychoeducation and coping skills, can reduce persistent psychological distress and physical problems and improve HRQoL [8].

As the number of patients surviving lymphoma continues to grow, interventions need to be easily accessible without increasing the burden on health services. Self-management interventions can be effective in strengthening the role of patients, by increasing patient engagement in care, and limiting the burden on health services [19, 20]. Self-management interventions aim to empower patients to have an active role in the management of their disease and its symptoms and consequences including treatment, physical, and psychosocial and lifestyle changes [21, 22]. Web-based technologies are particularly suitable for self-management interventions since they are easily accessible, can reach a large number of patients [19, 23], and provide more anonymity compared to face-to-face interventions [24]. Therefore, web-based interventions have the potential to eliminate barriers to psychosocial care for patients with cancer. However, it is important that such interventions should be evidence-based and empirically tested [25].

The Lymphoma InterVEntion (LIVE) trial consists of two interventions: (1) feedback to patients on their PROs, and (2) a web-based, self-management intervention named *Living with lymphoma*. Patients will be randomised to: (1) standard care, (2) standard care plus access to PRO feedback or (3) standard care plus access to PRO feedback and the Living with lymphoma intervention. PRO feedback enables patients to monitor their symptoms and compare them with outcomes among other patients. This may help to either reassure that what they experience is ‘normal’ or may empower them to take action. The *Living with lymphoma *intervention is based on CBT components and is an adaptation from the evidence-based BREAst cancer e-healTH (BREATH) intervention [26]. By using the *Living with lymphoma* intervention, patients will receive psychoeducation and learn coping skills which they can apply as self-management skills in daily life.

## Methods/design

### Objectives and hypotheses

The objective of the LIVE trial is to examine whether PRO feedback and the* Living with lymphoma *intervention will increase self-management skills and satisfaction with information and reduce psychological distress. In concordance with the stress-coping model of Lazarus and Folkman (1984), psychological adjustment after cancer is determined by the balance between stress and resources [18]. Therefore, it is hypothesised that patients with access to PRO feedback and/or the *Living with lymphoma *intervention will report increased self-management skills and satisfaction with information (greater resources available for coping), and lower levels of psychological distress compared to patients receiving standard care. Moreover, it is expected that patients with access to both PRO feedback and the *Living with lymphoma *intervention will benefit most.

### Study design

**Fig. 1:**
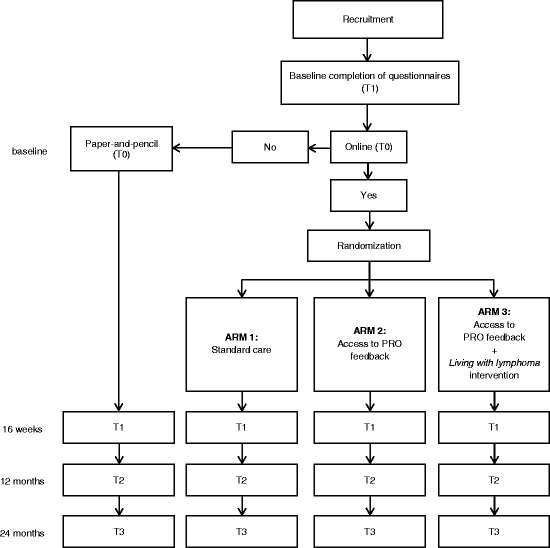
Overall study design of the Lymphoma InterVEntion (LIVE) trial

The LIVE trial is designed as a nonblinded randomised controlled trial with three arms. For an overview of the design of the trial, see Fig. 1. Standard care plus the access to PRO feedback and the *Living with lymphoma *intervention (arm 3) will be compared to standard care plus access to PRO feedback (arm 2) and standard care (arm 1). Patients with lymphoma from various hospitals in the Netherlands will be included and asked to complete questionnaires at four points in time: baseline (T0; 6 to 15 months after diagnosis), after 16 weeks (T1; post intervention), after 12 months (T2), and after 24 months (T3).

### Study population

All patients who have been diagnosed with HL or NHL, including chronic lymphocytic leukaemia (CLL), as defined by the International Classification of Diseases for Oncology-3 codes (ICD-O-3) [27], in the participating hospitals will be selected for participation via the Netherlands Cancer Registry (NCR). Patients must be aged 18 years or older at the time of diagnosis. Patients who have problems with the Dutch language, patients with severe psychopathology or dementia, and patients in transition to terminal care will be excluded from the study.

### Setting

The LIVE trial will be conducted within the Patient Reported Outcomes Following Initial treatment and Long-term Evaluation of Survivorship (PROFILES) registry [28]. PROFILES is a tool that enables data collection management; from inviting patients to participation in studies, to collecting PRO data via web-based or mailed questionnaires and linking these data with clinical data. Since this trial is embedded in the population-based PROFILES lymphoma registry, we have access to information on response, uptake, and user characteristics of patients with lymphoma in a web-based intervention.

### Recruitment

The population-based NCR of the Netherlands Comprehensive Cancer Organisation (IKNL) will be used to select all patients in the participating hospitals who meet the inclusion criteria. The NCR registers all newly diagnosed cancer patients within 6 months after diagnosis. After excluding deceased patients, the treating haemato-oncologists are asked to verify the patients’ study eligibility. All eligible patients will be invited for participation by their own haemato-oncologist. The haemato-oncologists will provide the eligible patients with an invitation package, including an invitation letter and leaflet to inform them about the study, a postcard, and two Informed Consent Forms (i.e. one for the researchers and one for the patient). The letter explains the study objectives and includes a link and password to a secure website so that patients can complete questionnaires online. If patients prefer paper-and-pencil participation, they can complete the postcard and return it by mail to the study manager. Patients will then receive paper-and-pencil questionnaires and a pre-stamped envelope within 1 week of receipt of the postcard. Patients are informed that paper-and-pencil participation automatically means that they will not be able to participate in the LIVE trial and only participate in the observational PROFILES lymphoma registry, as both PRO feedback and the Living with lymphoma intervention are web-based.

If the questionnaire is not completed within 3 weeks, a reminder will be sent by the treating haemato-oncologists. After obtaining informed consent, the subsequent communication to the patients will be addressed via PROFILES. To guarantee anonymity, questionnaires only contain a study number.

### Randomisation

Patients who complete the baseline questionnaire online and consent to participate in the LIVE trial will be automatically randomised in an equal ratio (1:1:1) to one of the three study arms: (1) standard care, (2) standard care plus access to PRO feedback or (3) standard care plus access to PRO feedback and the *Living with lymphoma *intervention. This randomisation will be performed using block randomisation. The randomisation will be performed by a computer randomisation program which will ensure a balance in sample size across groups over time [29].

### Interventions versus standard care

#### Arm 1: Standard care

For patients randomised to arm 1, the haemato-oncologist provides standard care. Most haemato-oncologists give their patients leaflets regarding the diagnosis and treatment they receive. Most information is given during the initial treatment phase, and some of the haemato-oncologists give additional information during follow-up for ad hoc referrals if needed by the patient. Patients who receive standard care can use information about lymphoma on the Internet, but do not have access to PRO feedback or the *Living with lymphoma *intervention.

#### Arm 2: PRO feedback

Patients randomised to arms 2 and 3 have access to PRO feedback, including general HRQoL, physical, emotional, cognitive and social functioning, fatigue, neuropathy (only for patients with high-grade NHL), and anxiety and depressive symptoms.

Patients can compare their scores to mean scores of other patients with lymphoma (same sex and age group) and/or a normative population (same sex and age group) to find out whether their scores are average or not (using a traffic light model). A detailed description of how to interpret the scores is added to assist patients in understanding the graphs. Mean scores of the lymphoma sample are extracted from data of our previous research on HRQoL among 856 patients with lymphoma [30]. The normative population was selected from a reference cohort of 1859 individuals from the general Dutch population (CentERpanel). This cohort is representative for the Dutch-speaking population in the Netherlands [31].

**Fig. 2:**
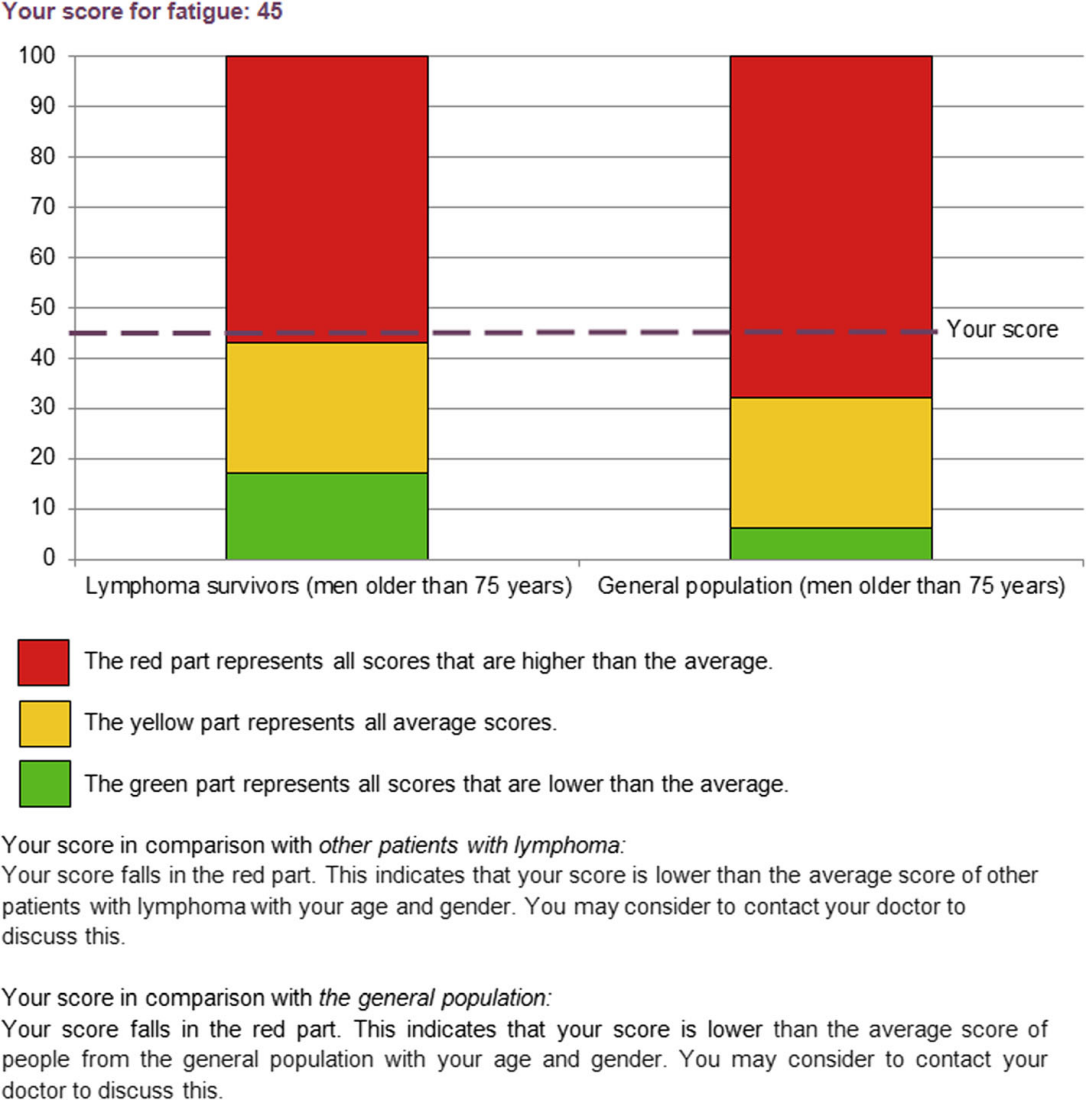
An example of patient-reported outcome feedback

Individual scores will be integrated into graphical displays with coloured bar-charts [32, 33]. The colours of the bar-charts are related to clinically relevant mean differences of the evidence-based guidelines of the EORTC QLQ-C30 [34]. A score that differs less than the minimal medium clinically relevant difference from the mean score is considered ‘average’ (amber). A score that differs as much as or more than the minimal medium clinically relevant difference from the mean score is considered ‘above average’ (green) or ‘below average’ (red). The interpretation of anxiety and depressive symptoms is according to the published scoring algorithm: 0–7 indicating no or mild symptoms (green), 8–10 indicating moderate symptoms (amber), and ≥11 indicating severe symptoms (red) [35]. Patients with a score in the red part of the chart are advised to contact their general practitioner. For an example of PRO feedback, see Fig. 2.

To review the PRO feedback, patients have to click on the ‘feedback’ tab after completing the questionnaire. Patients can decide not to review their PRO feedback as they prefer not to.

#### Arm 3: PRO feedback plus *Living with lymphoma* intervention

In addition to PRO feedback, patients randomised to arm 3 get access to the* Living with lymphoma* intervention. This web-based self-management intervention is an adaptation of the evidence-based BREATH intervention for breast cancer survivors [26, 36]. The content of the intervention is adapted to warrant its relevance for patients with lymphoma. Symptoms that are typically common in patients with lymphoma, such as neuropathy, infections, and infertility, have been added.

**Fig. 3:**
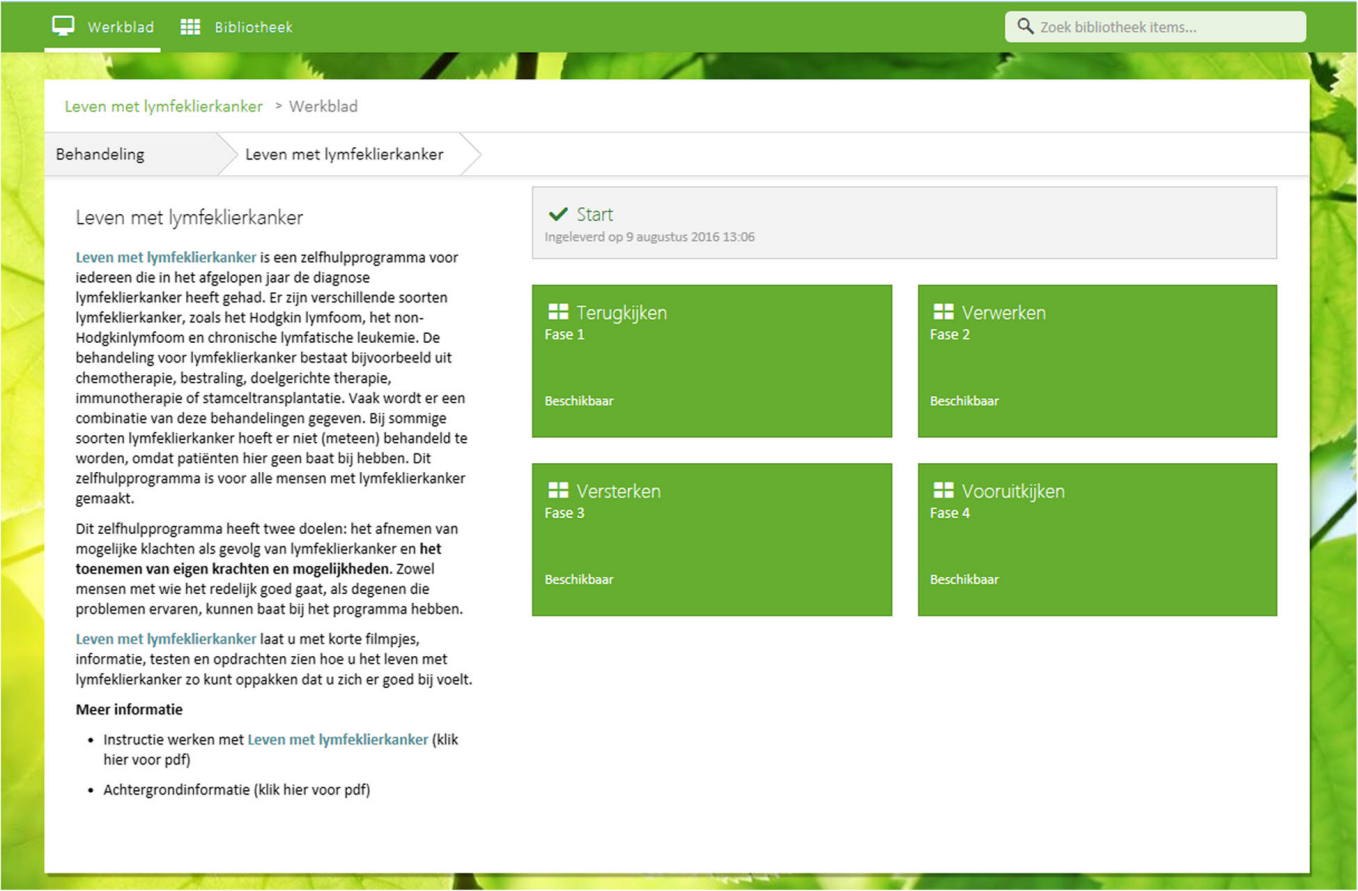
Screenshot from the ‘work space’ of the *Living with lymphoma* intervention with phase structure (in Dutch)

One key feature of the intervention is the ‘work space’ that includes four phases: (1) ‘looking back’, (2) ‘emotional processing’, (3) ‘strengthening’, and (4) ‘looking ahead’. For a screenshot of the ‘work space’, see Fig. 3. The intervention is based on CBT techniques, such as psychoeducation, to enhance patients’ knowledge and skills, for instance by providing tailored advices based on patients’ input. Working ingredients of the four phases include information, assignments, assessments, and videos. The information part provides patients with knowledge on various subjects such as adverse physical and psychological problems, work, sexuality, and lifestyle. Assignments are, for example, writing tasks, social engagement or conversation tasks and aim to increase skill-building [26]. Assessments include tests that could be used by patients as a screening instrument of potential problems and are followed by automated feedback. Videos are clips extracted from recorded interviews with patients with lymphoma.

**Fig. 4:**
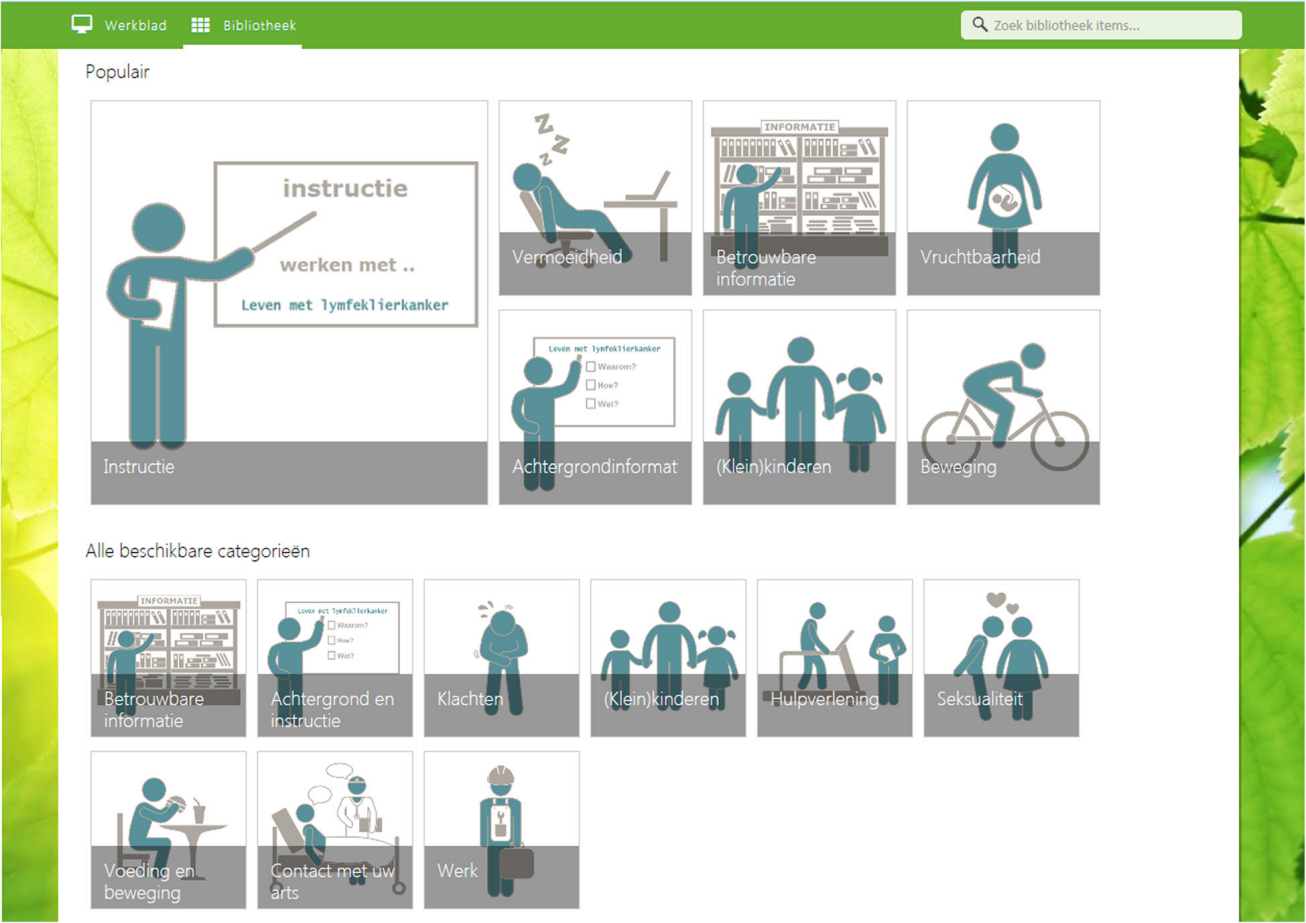
Screenshot from the library of the *Living with lymphoma* intervention (in Dutch)

Another feature of the intervention is the library with background and additional information on subjects from the four phases (e.g. work, sexuality, lifestyle). For a screenshot of the library, see Fig. 4. The library also contains links to additional health care services (e.g. psychologists, physiotherapists, dieticians).

The advised intervention usage is one part per week, with a duration of approximately 1 h. However, it is up to the patients how, and to what extent, they use the intervention. From the BREATH intervention it is known that patients use the website quite diversely [37]. The intervention is fully automated and nonguided and is delivered without the professional support of a therapist. Support for content or technical assistance is available by the study manager.

### Study outcome measures

Patient demographics and clinical information will be available from the NCR that routinely collects data on, among other things, patients’ age and sex, date of cancer diagnosis, histological classification, stage, treatment, and comorbidity. Information on marital status, educational level, and employment status are gathered by self-report using questionnaires.

#### Primary outcomes

*Self-management skills* are measured by the Health Education Impact Questionnaire (heiQ^TM^) [38]. The heiQ^TM^ contains 40 items across eight scales: positive and active engagement in life, health-directed activities, skill and technique acquisition, constructive attitudes and approaches, self-monitoring and insight, health service navigation, social integration and support, and emotional distress. Each item will be scored on a four-point Likert scale. The scale scores are obtained by computing the mean of respective items. Higher scores indicate better status or self-management, except for emotional distress, in which higher scores indicate higher distress [38]. The heiQ^TM^ has high construct validity [38]. Five scales of the heiQ^TM^ are validated among patients with cancer [39].

*Psychological distress* will be assessed by the 14-item Hospital Anxiety and Depression Scale (HADS) [35]. A sum score is obtained by adding the items. Its rating system is based on a four-point format and asks how the patient has felt in the past week. Higher scores indicate higher levels of psychological distress. The HADS has shown good reliability and validity in oncology settings [40, 41].

*Satisfaction with information* will be measured by an adapted version of the 9-item Information Satisfaction Questionnaire (ISQ) [42]. The ISQ has been widely used to assess overall information satisfaction and the need for involvement in decision-making. The original measure requires patients to categorise themselves into one of three groups: those who would like (1) all available information and to be involved in decisions about their illness, (2) only positive information about the illness, and (3) only limited information and would prefer the doctor to make the decisions. However, Fallowfield suggests that there is a distinction between the desire for information and involvement in decision-making [43]. Therefore, we divided that question into two items, one assessing the desire for information and one assessing the desire for involvement in decision-making. Patients are, furthermore, asked to rate their level of satisfaction with the information that they have received about their illness, treatment, and lifestyle. Each of these questions will be scored on a five-point Likert scale. The English version of the ISQ was translated into Dutch by forward-backward translation procedures. Questions about desire for more or less information, helpfulness of information, and the use of the Internet (to search for information) were added to the questionnaire.

#### Secondary outcomes

*Health-related quality of life―general* will be assessed using the Dutch validated European Organisation for Research and Treatment of Cancer (EORTC) Quality of Life Questionnaire (QLQ-C30) [44]. This 30-item questionnaire includes five functional scales, three symptom scales, a global health and quality of life scale, and several single-item symptom measures. All items will be scored on a four-point Likert scale, except for the global health and quality of life scale that is scored on a seven-point linear analogue scale. After linear transformation, all scales and single-item measures range in score from 0 to 100. Higher scores on functional and health and quality of life scales indicate better functioning or HRQoL, whereas higher scores on symptom scales indicate more symptoms.

*Health-related quality of life―lymphoma-specific* will be assessed with the EORTC disease-specific modules, i.e. QLQ-HL27 for HL, QLQ-NHL-HG29 for high-grade NHL and QLQ-NHL-LG20 for low-grade NHL, and QLQ-CLL17 for CLL [45]. The modules are divided into multi-item subscales including symptom burden, physical condition/fatigue, worries/fears, health and functioning, emotional impact, and neuropathy (only in the NHL-HG29 module). Items are scored on a four-point Likert scale. After linear transformation, all scales range in score from 0 to 100, whereby a higher score reflects more problems [45].

*Self-efficacy* with regard to symptoms (in this study as a result of lymphoma) will be measured with the Self-efficacy Scale (SE28) [46–48]. This scale consists of seven items, which will be scored on a four-point Likert scale. A higher score reflects a greater sense of control. This scale had previously been used to assess self-efficacy concerning post-cancer fatigue [49].

*Adjustment to cancer* will be assessed using the 40-item Mental Adjustment to Cancer Scale (MAC) [50]. Items are rated on a four-point Likert scale. The summary scales (i.e. summary positive adjustment scale, summary negative adjustment scale) can be used to identify general adjustment styles for cancer. The summary positive adjustment scale includes 17 items and scores range from 17 to 68 (cut-off ≥47), whereas the summary negative adjustment scale includes 16 items with scores ranging from 16 to 64 (cut-off ≥36) [51]. Summary scales are scored through addition of the items.

*Illness perceptions* will be assessed using the validated Brief Illness Perception Questionnaire (B-IPQ) [52]. This scale has nine items, measuring cognitive representations, emotional representations, and illness comprehensibility. Items are scored on a continuous linear 0–10-point scale. A higher score reflects a more threatening view of the illness. The B-IPQ has been previously cross-culturally adapted into the Dutch Language Version (Brief IPQ-DLV), with acceptable face and content validity [53].

*Fatigue* will be assessed with the 20-item validated Multidimensional Fatigue Inventory (MFI) [54]. The MFI covers five scales: general fatigue, physical fatigue, reduced activity, reduced motivation, and mental fatigue. Each scale contains four items, with two items formulated in a positive (e.g. ‘I feel fit’) and two formulated in a negative direction (e.g. ‘I feel fatigued’). All items are scored on a five-point Likert scale. The negatively formulated items must be recoded before adding up scores. Higher sum scores correspond to more acute levels of fatigue. The MFI is reliable and valid to assess fatigue in patients with cancer [54].

*Health care use* will be assessed by single items: ‘How often did you contact a general practitioner in the past 12 months?’, ‘How many of these visits were related to cancer of the consequences of your cancer?’, ‘How often did you visit a medical specialist in the past 12 months?’, ‘How many of these visits were related to cancer or to the consequences of your cancer?’ These questions were asked in a similar way as by Statistics Netherlands [55] (http://statline.cbs.nl/). Three questions are asked about follow-up appointments (whether or not receiving follow-up appointment, the frequency of follow-up appointments, and satisfaction with this frequency). Furthermore, patients are asked whether they visited a psychologist, psychiatrist or social worker and the last question was ‘Did you receive care after the treatment of your cancer?’ To answer this question, patients could either choose ‘No’ or ‘Yes’ and then choose multiple additional care services from a list: sexologist, pastoral care, dietician, physical therapist, oncological rehabilitation, creative therapy, oncology nurse, or contact with other cancer survivors.

#### Covariates

*Comorbidity* at the time of survey will be assessed with the adapted Self-administered Comorbidity Questionnaire (SCQ) [56]. Patients will be asked to identify comorbid conditions that have developed since diagnosis.

*Personality* will be assessed using the Big Five Inventory (BFI) [57]. The BFI is a 44-item inventory designed to measure the Big Five dimensions: extraversion, agreeableness, conscientiousness, neuroticism, and openness to experience. Items are scored on a five-point Likert scale. Scale scores will be created by averaging the items for each domain. The Dutch BFI has good psychometric quality [58].

#### Usage statistics

In addition to the standardised questionnaires, technical data on the use of the intervention, such as frequency, duration, and activity, will be evaluated.

### Sample size calculation

Sample size calculation was performed using G*Power version 3.1.9.2 for Windows. Based on the three primary outcomes of this trial, effect on patient level is defined as increased self-management skills or satisfaction with information (as measured by the heiQ^TM^ and the ISQ, respectively) or reduced psychological distress (as measured with the HADS). Therefore, effectiveness of LIVE is demonstrated when one of the three effects is statistically significant. Significance level of the sample size calculation was adjusted to *p* ≤ 0.167 to keep the overall chance for type-I errors at 5%.

Clinically important differences will be determined with Norman’s ‘rule of thumb’, whereby a difference of approximately 0.5 SD indicates a threshold of discriminant change in quality of life scores of a chronic illness [59]. To detect a clinically important difference with 90% power, a sample size of 222 patients with lymphoma (74 in each group) is needed. This sample size calculation is based on a medium effect size of 0.25 for repeated measures analysis of variance (ANOVA) with two measurements, since at least two measurements are necessary to compare preintervention and postintervention outcomes. We take into account a response rate of 70% as observed in earlier studies (of them 60% are expected to complete the questionnaires online) and a study dropout rate of 25%, based on a systematic review on adherence to Internet interventions for anxiety and depression [60]. This results in 663 patients with lymphoma who need to be invited for participation.

### Statistical analyses

All statistical analyses will be performed using Statistical Analyses Software (SAS; version 9.4 for Windows, SAS Institute Inc., Cary NC, USA). Analyses on effectiveness of the intervention will be primarily done according to intention-to-treat methodology. Second, per-protocol analysis will be performed to analyze the efficacy of the intervention. All statistical tests will be two-sided and considered significant if *p* < 0.05.

Missing outcome data will be assumed to be ‘missing at random’ (MAR), conditional on key predictors of ‘missingness’ (in particular, baseline values of the outcome variables of interest, and study arm).

Patients’ sociodemographic and clinical variables will be compared at baseline between the three study arms using chi-square analyses for categorical variables and ANOVA for continuous variables and will be analysed as covariates.

Repeated measures analysis using generalised estimating equations, which account for the intra-patient dependency of the repeated measures, will be used to analyse the effect of the intervention on the outcome variables. We will investigate differences in effect of the two intervention arms and the arm receiving standard care at the different time points. Differential effects of the intervention arms by age, cancer subtype, and baseline levels of the outcomes of interest will be assessed for the outcome measures by adding terms for the interaction between age, cancer subtype, baseline levels, and care arm to the regression models.

Routinely collected data from the population-based NCR on patient and tumour characteristics will enable us to compare paper-and-pencil respondents with online respondents, as well as respondents with nonrespondents and patients with unverifiable addresses in order to determine the external validity of the results and answer our second study objective.

## Discussion

Regular screening of symptoms by the use of PROs and access to resources for coping skills could help to detect and/or manage symptoms that up to a quarter of patients with lymphoma are experiencing.

The results of the LIVE trial will provide novel insights into whether access to PRO feedback and the *Living with lymphoma *intervention will be effective in increasing self-management skills and satisfaction with information, and reducing psychological distress. Since one third of patients will be randomised to solely access to PRO feedback and not to the *Living with lymphoma *intervention, it will be possible to investigate the superiority of access to PRO feedback as well as the superiority of access to PRO feedback and the *Living with lymphoma *intervention compared to standard care.

The LIVE trial is embedded in the population-based PROFILES lymphoma registry, which provides a unique setting to ascertain information on response, uptake, and characteristics of patients with lymphoma in web-based intervention(s). This information is important with respect to the generalisability of results and, moreover, it demonstrates which patient subgroups will benefit most from PRO feedback and the *Living with lymphoma *intervention*.* Patients will not be selected based on their symptoms or distress level prior to study entry and it is up to patients themselves how, and to what extent, they use the intervention(s). When effective, access to PRO feedback and the *Living with lymphoma *intervention could serve as easily and widely accessible interventions for coping with lymphoma in the Netherlands.

### Trial status

Recruiting.

## Additional files

Additional file 1: SPIRIT 2013 Checklist: recommended items to address in a clinical trial protocol and related documents. (DOC 120 kb)

Additional file 2: SPIRIT Figure: Schedule of enrolment, interventions, and assessments. (DOC 66.5 kb)

## Abbreviations

BREATH: BREAst cancer e-healTH
CBT: Cognitive behavioural therapy
CLL: Chronic lymphocytic leukaemia
HL: Hodgkin lymphoma
HRQoL: Health-related quality of life
ICD-O-3: International Classification of Diseases for Oncology, third edition
IKNL: Netherlands Comprehensive Cancer Center
LIVE: Lymphoma InterVEntion
MAR: Missing at random
NCR: Netherlands Cancer Registry
NHL: Non-Hodgkin lymphoma
PRO: Patient-reported outcome
PROFILES: Patient Reported Outcomes Following Initial treatment and Long-term Evaluation of Survivorship
SAS: Statistical Analyses Software
