# Supplementary material for: Lymphoma InterVEntion (LIVE) – patient-reported outcome feedback and a web-based self-management intervention for patients with lymphoma: study protocol for a randomised controlled trial
Source: Trials. 2017 Apr 28;18:199. doi: 10.1186/s13063-017-1943-2 (PMC5408371; doi:10.1186/s13063-017-1943-2)
Supplement: Supplementary file 2 — SPIRIT Figure: Schedule of enrolment, interventions, and assessments. (DOC 66.5 kb) [file 13063_2017_1943_MOESM2_ESM.doc]

**Additional file 2. SPIRIT Figure: Schedule of enrolment, interventions, and assessments**

|  |  | **STUDY PERIOD** | | |
| --- | --- | --- | --- | --- |
|  | **Enrolment Allocation** | **Post-allocation** | **Follow-up** | |
| **TIMEPOINT*** | ***T_0_*** | ***T_1_*** | ***T_2_*** | ***T_3_*** |
| **ENROLMENT:** |  |  |  |  |
| Eligibility screen | X |  |  |  |
| Informed consent | X |  |  |  |
| Allocation | X |  |  |  |
| **INTERVENTIONS:** |  |  |  |  |
| PRO feedback |  |  |  |  |
| PRO feedback + *Living with lymphoma* intervention |  |  |  |  |
| **ASSESSMENTS: **** |  |  |  |  |
| **Sociodemographic variables** | X | X | X | X |
| **Primary outcomes** |  |  |  |  |
| Satisfaction with information (ISQ) | X | X | X | X |
| Self-management skills (heiQ) | X | X | X | X |
| Psychological distress (HADS) | X | X | X | X |
| **Secondary outcomes** |  |  |  |  |
| General health-related quality of life (EORTC QLQ-30) | X | X | X | X |
| Lymphoma specific health-related quality of life― (QLQ-HL27, NHL-HG29, NHL-LG20, CLL17) | X | X | X | X |
| Self-efficacy (SE-28) | X | X | X | X |
| Adjustment to Cancer (MAC) | X | X | X | X |
| Illness perceptions (B-IPQ) | X | X | X | X |
| Fatigue (MFI) | X | X | X | X |
| Health care use | X | X | X | X |
| **Covariates** |  |  |  |  |
| Comorbidity (SCQ) | X | X | X | X |
| Personality (BFI) | X | X | X | X |
